# Biofabrication of tissue engineering vascular systems

**DOI:** 10.1063/5.0039628

**Published:** 2021-05-07

**Authors:** Qiao Zhang, Èlia Bosch-Rué, Román A. Pérez, George A. Truskey

**Affiliations:** 1 Department of Biomedical Engineering, Duke University, Durham, North Carolina 27708, USA; 2 Bioengineering Institute of Technology (BIT), Universitat Internacional de Catalunya (UIC), Sant Cugat del Vallès 08195, Spain

## Abstract

Cardiovascular disease (CVD) is the leading cause of death among persons aged 65 and older in the United States and many other developed countries. Tissue engineered vascular systems (TEVS) can serve as grafts for CVD treatment and be used as *in vitro* model systems to examine the role of various genetic factors during the CVD progressions. Current focus in the field is to fabricate TEVS that more closely resembles the mechanical properties and extracellular matrix environment of native vessels, which depends heavily on the advance in biofabrication techniques and discovery of novel biomaterials. In this review, we outline the mechanical and biological design requirements of TEVS and explore the history and recent advances in biofabrication methods and biomaterials for tissue engineered blood vessels and microvascular systems with special focus on *in vitro* applications. *In vitro* applications of TEVS for disease modeling are discussed.

## I. INTRODUCTION

Cardiovascular diseases (CVDs) are the leading cause of death in the United States^1^ and many developed countries. In 2016, the prevalence of CVD in adults with age ≥20 in the US is 48% (Ref. 2) and the risk of CVD increases with age.^3^ The underlying cause for most CVD is the progression of atherosclerosis in which plaques form on the inner wall of arterial lumen known as the intima. Accumulation of plaque can obstruct blood flow, and plaque-induced thrombosis or embolism can cause blockage of the vessel and eventually lead to ischemia. Rupture of the plaque results in thrombus formation, which leads to ischemia, heart attack, or stroke.^4^

Risk factors including smoking, obesity, diabetes, high blood pressure, and high cholesterol result in the accumulation and modification of low-density lipoprotein (LDL).^4–6^ These changes lead to the activation of endothelial cells and subsequent recruitment of circulating monocytes, which later differentiate to macrophages and promote foam cell formation. While the details of disease development are well known, the interaction of various genetic factors is poorly understood. To develop precision medicine for vascular disease, effective models must be available to recapitulate the features of the disease, assess the role of genetic factors, and provide correct treatment responses.

Approaches to mitigate the impact of CVD include preventative measures, such as diet, exercise, medication to treat the underlying causes, and measures applied after symptoms appear. Preventive measures are often applied after symptoms appear to reduce the likelihood of subsequent events. Current strategies for treating symptoms are to restore the blood flow by using medications to clear the plaque or surgical procedures such as angioplasty and stent placement, endarterectomy, and bypass.

Among the current strategies to treat CVD, coronary artery bypass grafting (CABG) is the most commonly performed procedure (79/100 000 inhabitants in the United States in 2010).^7^ While these approaches are effective at treating coronary heart disease, success in treating peripheral atherosclerosis, particularly in the legs, is less effective. Given these results, there is an urgent need for developing novel fabrication methods for replacement with grafts. The need for suitable grafts for bypass procedures and dialysis access is very high. Although autologous grafts, such as saphenous veins (SVs) or internal mammary arteries, provide the best clinical outcomes, there are many challenges including the availability of arteries and veins, mismatch of sizes, patient's medical conditions, and invasive procedures to harvest the graft. Vascular conduits made of synthetic polymers such as polytetrafluoroethylene (PTFE)/Teflon, expanded PTFE/Gore-Tex and polyethylene terephthalate/Dacron are suitable alternatives for replacing vessels with diameter >6 mm.^8^ For instance, expanded polytetrafluoroethylene (ePTFE) has shown great successes when used as grafts for lower limb bypass.^9^ While synthetic grafts are successful when used as large vessels (>6 mm), they present some problems when used as small diameter vessels (<6 mm), mainly related to low patency rate and thrombus formation. Therefore, the success rate is low when synthetic grafts are used in peripheral vessels and arteriovenous (AV) fistulas. The need for small diameter vessels has driven the discoveries of new materials and fabrication methods for tissue engineered vascular conduits.

Because of the ability to examine the effect of individual genes, murine models are commonly used to study atherosclerosis and other cardiovascular diseases.^10^ Animal models provide systematic information about the diseases and treatment response, the effect of multiple-cell types, organs, and systems (metabolism, immune, etc.).^11^ At the same time, murine systems possess many shortcomings and cannot recapitulate features of the pathology found in humans. Wild type mice have different cholesterol and lipoprotein metabolism and transport mechanisms, using high density lipoprotein (HDL) to transport cholesterol, while human uses low density lipoprotein (LDL).^12^ In addition, hemodynamic conditions are very different compared to human as mice have smaller blood vessels and higher heart rate.^13^ Although genetically modified murine models have been developed to overcome some of the shortcomings, the treatment responses tested in murine models cannot be directly translated into the human subject.^11,14^

A promising alternative to the use of animal models is tissue-engineered vascular systems (TEVS), which can be used to replace damaged arteries *in vivo*, serve as arteriovenous fistulas for dialysis patients, and promote revascularization of damaged tissue. TEVS with human cells can recapitulate key features of CVD *in vitro*, accurately predict the treatment responses to human cells and tissue, and can be used to examine the role of various genetic factors identified in association studies which are poorly understood due to lack of suitable model systems. Biofabrication techniques have been widely used to manufacture TEVS for both *in vivo* and *in vitro* purposes. As TEVS for *in vivo* applications have been thoroughly reviewed in many studies,^15–18^ we will focus on the *in vitro* application of TEVS. In this review, we will briefly summarize the history and development of TEVS and the design criteria. We will introduce the applications of biofabrication techniques and materials used for fabricating both tissue-engineered blood vessels (TEBV) and microvascular systems (MVS). Finally, we will discuss their use as disease model systems.

### A. Human vascular system

**FIG. 1.**
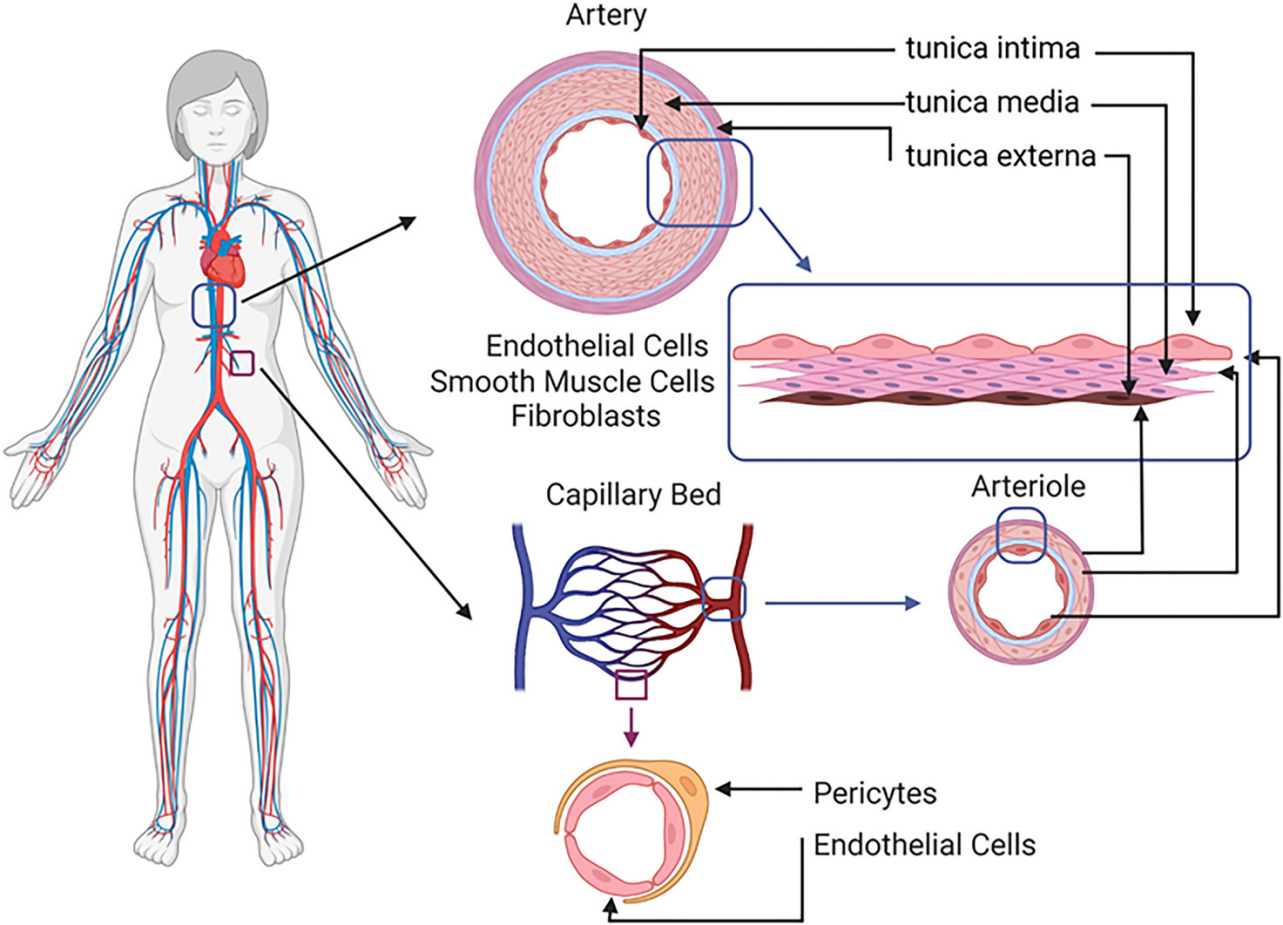
Blood circulation system and the cross-section images of each type of vessel human blood circulation system which starts from heart pumping blood through artery, arteriole, capillary bed, venule, and eventually back to the heart through vein. Artery (red) and vein (blue) contain tunica intima with ECs, basement membrane containing extracellular matrix (ECM) proteins, tunica media with SMCs, and tunica externa with fibroblasts. Arteriole and venule are smaller than artery and vein, respectively, with thinner walls. Capillary bed is formed by network of capillaries with ECs and pericyte, which are the smallest vessels in the body and function as networks to supply necessary materials for organ metabolism (figure created with BioRender.com).

TEBVs and MVSs model large/medium size arteries and capillary networks, respectively. The common structures shared between arteries and veins are the lumen and three layers of wall structures: the tunica intima, tunica media and tunica externa (Fig. 1). The tunica intima is the innermost layer that is in contact with the blood and composed of a confluent layer of endothelial cells on a basement membrane. The endothelium acts as a physical barrier, limiting the transport of cells and molecules into tissue and regulating coagulation and inflammation. The endothelial basement membrane contains collagen IV, laminin isoforms, and fibronectin.^19^ Its composition varies with tissue type, and it regulates endothelial function and mechanosensitivity.^19,20^ The tunica media contains vascular smooth muscle cells with collagen, proteoglycans, and varying amounts of elastin-rich elastic fibers. This layer is responsible for vasoactivity through contraction and relaxation of smooth muscle cells. The adventitia (tunica externa) is comprised of loose connective tissue with fibroblasts, collagen, and a capillary network which provide nutrients and oxygen.

Collagen and elastin are the two major extracellular matrix components, which each contribute differently to the mechanical properties of the vessel wall. The vessels do not follow Hooke's law, but behave as non-linear elastic materials. Collagen mainly contributes to the high stress region (non-linear region), and elastin contributes to the low stress linear region of the stress–strain curve.^21^ Proteoglycans containing negatively charged glycosaminoglycan (GAG) interact with collagen to affect collagen mechanical properties and fibril formation.^22^ Removal of GAGs causes straighter collagen fibers and early recruitment of elastin and collagen fibers at lower strain, which results in an early transition point to the non-linear region of the stress–strain curve.^23^ GAGs also play a key role as anticoagulants.^24,25^

In the MVSs, capillary networks supply oxygen, nutrients, and hormones from blood for organ metabolism. Capillaries consist of a single endothelial layer (5–10 *μ*m diameter) surrounded by pericytes, which maintain the vessel structure. New capillaries are formed by vasculogenesis and angiogenesis, which are induced by interstitial flows and with *in vitro* growth factor supplementation.^26,27^

Given the different structures and purposes served by the two distinct vascular systems, the design criteria and material and methods used for these systems are different. In this section below, we will first briefly discuss the cell source used for both of the systems, and then we will review the design criteria, material and methods used, and disease modeling applications of each system.

### B. Cell sources

For implantation, the cells must be autologous to prevent rejection of the TEVS. *In vitro* studies often use the same donor for endothelium and medial cells, particularly if genetic diseases are studied. Autologous endothelial cells can be obtained from adipose tissue which is readily available from many individuals. The main disadvantage is that, as they are primary cell lines, they lose their proliferation potential after several passages in culture. In contrast, endothelial cells derived from circulating endothelial colony forming cells (ECFCs) have an extensive proliferative capacity and exhibit many of the functions of vascular endothelium.^28^ While ECFCs can be relatively easily isolated from peripheral blood, obtaining vascular smooth muscle cells (SMCs) with high proliferative potential and contractility remains challenging. SMCs show great variation in functional properties due to the origin from embryogenesis and the stage of differentiation.^29,30^ Vascular SMCs are primarily isolated from arterial and venous tissues,^31^ which require invasive procedures to harvest these cells, and at the same time, they have limited availability and proliferation potential.

The groundbreaking discovery of human induced pluripotent stem cells (iPSCs) provides a method to obtain tissue-specific cells.^32^ iPSCs can be easily obtained by transforming various adult cell types into pluripotent state, including dermal fibroblast or blood cells. iPSCs have the potential to proliferate and can be differentiated into cells from all three germ layers, including ECs and SMCs for TEVS applications.^33,34^ However, there are some pitfalls with the *in vivo* use of cells differentiated from iPSCs, which include their tumorigenic potential and immature differentiation. Although progress have been made to develop more mature somatic cells, for example, by endogenous expression of genes during differentiation instead of forced cDNA overexpression,^35^ and reduce the risk of tumorigenicity by using non-integrating methods,^36–39^ more effort is needed to ensure the safety of using iPSC cells for transplantation.

Many studies have successfully generated ECs and SMCs for vascular engineering through transdifferentiation of adult cells such as fibroblasts.^40–46^ Transdifferentiation can be induced by the expression of key genes that regulate EC or SMC differentiation, by small molecules or clustered regularly interspaced short palindromic repeats (CRISPR) technologies.^40,43,44,47,48^ ECs can be generated by the activation of toll-like receptor 3 and by the overexpression of ETV2, which is a critical transcriptional factor in the development of ECs.^43,44^ The functionality of these ECs improved limb perfusion and neovascularization after implantation in ischemic murine models. Alternatively, SMCs can be obtained through culturing partially differentiated pluripotent stem cells from fibroblasts on collagen IV coated surface.^45^ These cells exhibited SMC-like morphology and expressed SMC markers including smooth muscle actin (SMA), calponin and SM22.

To fabricate a TEBV, smooth muscle cells are mixed with ECM gel (collagen, fibrin, etc.)^49^ or cultured for a long period to form a dense cell sheet^50^ and then shaped to a tubular structure as tunica media. To endothelialize TEBVs, the inner lumen of the tubular structure formed by SMCs is filled with EC suspension. Then the two ends of the tube are sealed, and the tube is kept in the incubator for at least 45 min with frequent rotation to allow uniform attachment of ECs on the wall.^49^

## II. LARGE/MEDIUM VESSELS

### A. Design criteria for TEVS

To function properly *in vivo*, TEBV must be non-thrombogenic, not induce an inflammatory response or immune rejection, promote vascularization, and integrate with the host tissue. At the same time, they should have appropriate mechanical properties to resist arterial blood pressure while having the compliance to distend and relax during the cardiac cycle.

#### 1. Biological properties

One of the major causes for early graft failures is acute thrombosis;^51^ hence, special attention should be taken for the biomaterial choice. Collagen and many synthetic polymers used in vascular grafts are prothrombogenic. While endothelialization is often necessary for long term *in vivo* function of tissue engineered vascular grafts (TEVGs), TEVGs implanted in the arterial circulation often do not need an endothelial layer because the blood flow rates limit platelet adsorption.

Endothelialization of synthetic grafts and TEBVs represents one approach to regulate coagulation and avoid thrombosis. Luminal TEBV surface modifications, for example, with fibronectin, laminin, or fibronectin derived peptides (RGD or Arg-Gly-Asp), promote endothelial cell adhesion, proliferation, and migration. However, platelets and immune cells also bind to these molecules, limiting their usefulness. In this regard, several molecules have been identified that specifically bind to ECs, such as LXW7, an integrin αvβ3 ligand, which has a strong affinity to ECFCs and ECs, promoting rapid endothelialization.^52^ In a recent study, poly(2-methacryloyloxyethyl phosphorylcholine-co-methacrylic) association with the peptide sequence arginine, glutamate, aspartate, and valine (REDV) or hemocompatible peptide-1 (HCP-1, HGGVRLY) could also enhance human umbilical vein endothelium (HUVEC) capture from blood and exhibit anti-platelet properties.^53^ Alternatively, matrix metalloproteinases (MMP) have been shown to be heavily involved in vessel re-endothelialization to modulate endothelial cells adhesion, proliferation, and migration.^54^

Tissue integration determines the long-term success of implanted grafts.^51^ Host integration starts with host EC attachment, migration, and interaction with graft ECs to form a microvasculature. Then host SMCs migrate into the grafts, proliferate, and secrete ECM proteins such as elastin and collagen to support the long-term mechanical properties of implanted graft. Hence, TEBVs must allow adequate cell adhesion and migration for successful integration. Another important contributor to tissue integration is the material degradation time. Ideally, the implanted materials will be degraded and replaced by newly formed host tissue. Therefore, the degradation rate should match the regeneration rate, allowing the migration of vascular cells and the formation of new ECM.

#### 2. Mechanical properties

The implanted TEBV must be strong enough to withstand the pulsatile pressure created by the flow and have good compliance to resist plastic deformation. The compliance of internal mammary artery is around 11.5%/100 mm Hg.^55^ The thin surgical sutures connecting the TEBV to the native vessel could create high stresses at the anastomosis, so the grafts must have good suture retention strength. The suture retention strength measures the minimal pulling force that breaks a vessel by pulling on the suture attached to the vessel. The suture retention strength of internal mammary artery is around 1.3 N,^55^ which is a good reference for designing new TEBVs.

The burst pressure, $P_{\mathrm{burst}}$, is a common measure of the strength of a TEBV and is defined as the minimum pressure that causes the burst of a vessel. Since the burst pressure depends on the vessel diameter and wall thickness, the ultimate tensile pressure (UTS) of the vessel is calculated using the law of Laplace. The burst pressure of TEBVs is expected to be at least similar to native vessels, 3200 mm Hg and 1600 mm Hg, described for internal mammary artery and saphenous veins, respectively.^55^

Blood vessels are exposed to a wide range of stresses and strains and behave as non-linear elastic materials, typically expanding 10%–15%. For non-linear elastic material, the incremental modulus is the local slope of the stress–strain curve. Typical mechanical properties of native vessels have been summarized.^56^

### B. Materials for TEBVs

The material must closely resemble the structural features of native vessels and possess strong enough mechanical properties to withstand pressures. At the same time, these materials must be non-thrombogenic, non-immunogenic and allow host-tissue integration. Both synthetic and biological materials have been used to fabricate TEBV. Biostable synthetic materials, such as PTFE and Dacron, are commonly used as alternatives to saphenous veins (SVs) for large vessel bypass procedures. However, they have poor patency rates, and mechanical properties do not match with those of native blood vessels.^57^ In contrast, biodegradable materials slowly degrade in the body and are replaced by neoregenerated tissues.

#### 1. Synthetic materials

Polycaprolactone (PCL) is a hydrophobic polymer made by ring-opening polymerization of ϵ-caprolactone. PCL has a long degradation time, about 1–2 years by hydrolysis.^57^ TEBVs made of PCL material achieve a burst pressure around 3300 mm Hg and allow nearly complete reendothelialization.^58^ PCL grafts have been tested in many animal models.^58–60^ Besides pure PCL grafts,^61^ PCL can be combined with collagen,^62–64^ herapin,^65,66^ elastin,^67^ chitosan,^68^ and polyurethane^69,70^ to form TEBVs. For instance, three-layered vessels with different ratios of PCL-collagen-elastin in each of the layers exhibit an elastic modulus and compliance similar to native arterial vessels.^67^

Polylactic acid (PLA) is another hydrophobic polymer synthesized by direct polymerization of lactic acid or through ring-opening polymerization of lactide. It degrades slowly (∼1 year) through hydrolysis *in vivo*, producing lactic acid.^71,72^ TEBVs can be fabricated by seeding cells on the electrospun PLA sheet which were then rolled around a 0.7 mm-mandrel to form tubular vessels.^73^ These vessels permit host EC and SMC adhesion and achieved complete endothelialization after 2 months post implantation into rats. PLA has been blended with PCL in different ratios, allowing the control of mechanical properties and degradation rate,^74^ and achieving a burst pressure around 1700 mm Hg. After implantation of PLA TEBVs, it was replaced with neoartery that shared similar biological and mechanical properties of native arteries.^71^

Polyglycolic acid (PGA) is a hydrophilic polymer synthesized by polycondensation of glycolic acid or ring-opening polymerization of glycolide. This quick degradation time of 2 to 4 weeks^75^ does not allow enough time for neotissue regeneration. Hence, PGA scaffolds are often seeded with cells or blended with other materials. For instance, mature vessels are obtained by seeding SMCs isolated from the medial layer of bovine aorta into tubular PGA scaffolds and then perfused in a bioreactor that applied pulsatile stress to the vessel,^76^ achieving a burst pressure higher than 2000 mm Hg. As another example, PGA was used as the core and PLA as a sheath to form a two-layer graft.^77^ Three-layered blood vessels are produced by wrapping PGA and PCL sheets seeded with SMCs, and PGA sheets seeded with fibroblasts around silicone tubes,^78^ and ECs formed the luminal surface. The overall manufacturing time for this scaffold is around 1.5 months. The TEVGs show great coverage of ECs and the ultimate strength is close to native arteries.^67^ Grafts made of PGA have been tested in many animal models.^77,79,80^

While TEBVs made with synthetic materials (Table I) show promising results *in vivo*, they have the following disadvantages: (1) lack of cell–ECM interactions to facilitate cell attachment, migration and remodel matrix, (2) lack of cleavable sites for MMPs (cell adhesion proteins or peptides and MMP cleavages sites could be added or coated to some of these polymers by modifying surface properties), (3) lack of typical three-layer vessel structure, (4) mismatch of mechanical properties, and (5) lack of vasoactivity. Furthermore, degradation of materials such as PGA and PLA results in an acidic local environment, which adversely influences the proliferation and biological functions of nearby cells.^81,82^ The long fabrication or maturation time for cells to generate ECMs and replace synthetic materials times limits the applications of these TEBVs for *in vitro* disease modeling.

**TABLE I.** Properties of synthetic materials.

Material	Degradation time	Burst pressure	Allow host cell integration	Small/large animal study	Require prior cell seeding	Main biofabrication method	Fabrication time before implantation	*In vitro* disease modeling
Synthetic material								
PCL	1–2 years^57,74^	3300 mmHg (Ref. 58)	Yes	Yes^58,59^/Yes^60^	No	Electrospinning	1 day	No
PLA	∼1 year^71,72,74^	NA	Yes	Yes^73^/No	Yes	Electrospinning	2 days	No
PLCL	∼1 year^74^	∼1700 mmHg (Ref. 71)	Yes	Yes^71^/No	No	Electrospinning	1 day	No
PGA	∼1 month^75^	>2000 mmHg (Ref. 76)	Yes	Yes^79^/Yes^77,80^	Yes	Mold shaping, sheet rolling	>7 weeks	No

#### 2. Biological materials

Biological materials are naturally present in the human body or in other organisms and mostly consist of ECM proteins (Table II). Many different biological materials used for fabricating TEBVs include collagen, fibrin, silk fibroin, chitosan, and alginate.

**TABLE II.** Properties of biological materials. NA: Not available.

Material	Burst pressure	Allow host cell integration	Small/large animal study	Require prior cell seeding	Main biofabrication method	Fabrication time before implantation	*In vitro* disease modeling
Natural material							
Collagen	1600 mmHg (Ref. 93)	NA	Yes^91^/No	No	Mold shaping	1 day	Yes^93,171,174^
Fibrin	3100 mmHg (Ref. 100)	Yes	Yes^97^/Yes^98–100^	Yes	Mold shaping	>5 weeks	No
Silk fibroin	2800 mmHg (Ref. 108)	Yes	Yes^103–105^/Yes^106,107^	No	Sheet rolling electrospinning	1 day	No
Chitosan	4000 mmHg (Ref. 113)	NA	Yes^110^/Yes (short time of 3 days)^110^	No	Extrusion mold shaping	1 day	No

Collagen is the major ECM protein in the tunica media and tunica externa of native blood vessels and, therefore, is a preferred choice for fabricating TEBVs. Collagen can be categorized into fibrillar and non-fibrillar collagen,^83^ being fibrillar collagens extensively studied as biomaterials due to their mechanical properties. Each collagen molecule is formed by three polypeptide chains, which may be identical or different. Many collagen molecules aggregate and align to form collagen fibrils.^84^ The microscopic structures and concentration of fibrillar collagens determine the mechanical strength of the macroscopic collagen based biomaterials.^85^ The first TEBV made with collagen gel required Dacron as structural support^86^ due the poor mechanical strength.

Efforts to improve the mechanical strength of collagen include mechanical conditioning of the collagen matrix by applying cyclic strain^87,88^ or by mixing with other materials like elastin.^89^ The geometry, density, and extent of fibril alignment of dense collagen gels can also be controlled through gel aspiration–ejection process.^90^ To increase the collagen density, Li *et al.* prepared 1 mm inner diameter collagen tubes with overnight dehydration followed by cross-linking with genipin, producing burst pressure around 1300 mm Hg.^91^

Another way to increase the mechanical strength is through plastic compression, which reduces the water content of collagen gel to increase fiber density.^92,93^ Plastic compression of an annular collagen matrix seeded with human neonatal dermal fibroblasts or human bone marrow derived mesenchymal stem cells yielded 800 *μ*m diameter TEBVs with burst pressure around 1600 mm Hg, which is similar to veins although the Young's modulus (70 kPa) and ultimate tensile strength (∼110 kPa) are lower than native vessels.

Fibrin is a viscoelastic polymer formed by polymerization of the plasma protein fibrinogen through thrombin-catalyzed cleavage of two pairs of peptides in the central nodule of fibrinogen monomer.^94^ Fibrin stimulates the production of ECM proteins. More specifically, fibroblasts and SMCs produce more collagen when seeded in fibrin gels compared to collagen ones.^95,96^ In accordance with these results, TEBVs fabricated with fibrin gels seeded with SMCs also promote collagen synthesis and achieve mechanical properties (burst pressure of 3164 mm Hg and 2 N suture retention) similar to those of rat abdominal aorta and human internal mammary artery.^97^ Grafts made of fibrin have been tested in both small and large animal models.^97–100^ Because fibrin-based TEBVs require seeded cells to generate ECM, the overall fabrication time can last for months, which presents as an obstacle for *in vitro* applications.

Silk fibroin is produced by silkworm or spider and has been used in biomedical applications like sutures, presenting great biocompatibility and mechanical properties.^101^ The surface of silk fibroin can be easily modified because of the availability of amine and acid functional groups on the side chains. The *in vivo* degradation time of silk fibroin ranges from several months to 1 year,^102^ which makes silk fibroin suitable for fabricating TEBVs.^103–107^ Regarding the mechanical properties, silk fibroin based grafts achieve 2800 mm Hg burst pressure without prior cell seeding.^108^

Chitosan is a polysaccharide produced by shell-fish through deproteinization–demineralization–decolorization–deacetylation.^109^ The biodegradability, biocompatibility and easily modifiable properties of chitosan make it a promising candidate for tissue engineering applications and TEBVs made od chitosan have been tested with animal models such as rat and sheep.^110^ Chitosan is degraded by enzymes such as lysozyme, releasing non-toxic oligosaccharides.^111^ The Young's modulus of small (6 mm) chitosan conduit tubes ranges from 5 to 7 MPa, depending on the concentration used.^112^ TEBVs made by coating chitosan/gelatin on both inside and outside of fiber-based knitted chitosan tube have burst pressure around 4000 mm Hg with high suture-retention, enabling the spread and growth of SMCs.^113^ Given the short degradation time (several days) of chitosan,^114^ it is difficult for neotissue formation after implantation. Hence, chitosan is often combined with PCL to enhance the mechanical strength and cell attachment of PCL.^68,115–120^

Alginate is an anionic polymer typically extracted from brown algae consisting of linear co-polymers of (1-4)-linked β-d-mannuronic acid (M) and α-l-guluronic acid (G) monomers.^121^ These monomers can be distributed as consecutive M residues, consecutive G residues, or alternating MG residues. Higher content of G residues and higher molecular weight results in an enhancement of mechanical properties and slow degradation *in vitro.*^122^ The most striking property of alginate is its ability to rapidly cross-link when in contact with divalent ions, such as calcium. Divalent cations bind to G monomers of one polymer chain and with G monomer of the adjacent polymer chain, resulting in a hydrogel structure, known as egg-box model of cross-linking. The resulting hydrogel is similar to extracellular matrices in terms of the water content, and together with its biocompatible and quick cross-linking properties makes it attractive for tissue engineering applications.

The main disadvantage of alginate is its short-term stability when in contact with physiological fluids as it can be dissolved due to exchange of calcium by sodium present in higher concentrations in cell culture media or physiological fluids, weakening its structure. In this regard, TEBVs composed of 4% alginate presented low ultimate strength (0.18 MPa)^123^ or could not be anastomosed when implanted *in vivo*^124^ although it demonstrated successful re-vascularization of ischemic limb.

### C. Biofabrication methods for TEBV

#### 1. 3D bioprinting

3D bioprinting, also known as additive manufacturing, gained interest in the last decades due to the ability to develop customizable and personalized organ-like products. The process involves a computer-aided layer-by-layer deposition of cells and biomaterials, known as bioinks, to develop 3D structures. A distinctive feature of 3D bioprinting is the ability to fabricate a wide range of tubular structures and sizes, from artery to small arteriole-size tubular structures, as well as branched structures or even incorporating pre-vascularized networks within 3D scaffolds for tissue regeneration. The bioink must not affect cell viability and should have the ability to be transformed from liquid to a gel or solid state with proper mechanical properties to maintain the structure once printed.^125^ Alginate is a widely used bioink due to its ability to rapidly cross-link, although other hydrogels such as collagen, gelatin, fibrin and their combination have been employed for vascular 3D printing. Extrusion-based and inkjet printing have been used to develop TEBVs, whereas laser-assisted 3D bioprinting has been applied to develop MVS.^126^

**FIG. 2.**
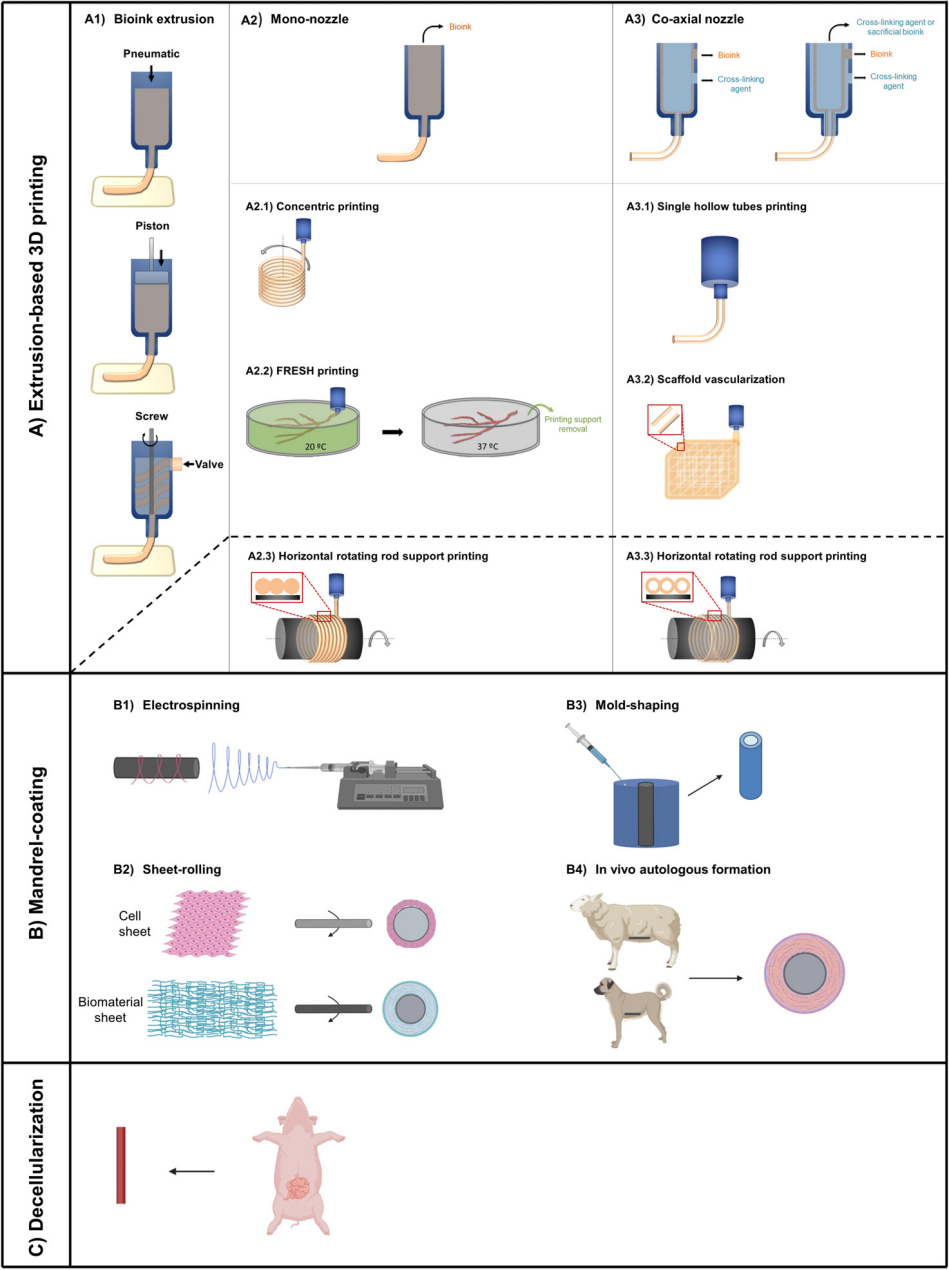
Biofabrication methods for vascular systems. (a) Extrusion-based 3D printing strategies for tubular vessel manufacturing: (a1) extrusion 3D printing uses pneumatic or mechanical (piston or screw) forces to extrude continuous material; (a2) using mono-nozzle print head, tubular vascular structures can be obtained through (a2.1) concentric vertical printing; (a2.2) using freeform reversible embedding of suspended hydrogels (FRESH) and (a2.3) horizontal printing on the surface of a rotating rod. (a3) Using co-axial nozzles as the print head, (a3.1) hollow tubular structures can be obtained directly, (a3.2) if organized in 3D structure, vascularized scaffolds can be developed and (a3.3) if printed on the surface of a horizontal rotating rod, a tubular structure with microchannels on the vessel wall can be obtained. Other strategies to form tubular graft structures are mandrel-coating based: (b1) electrospinning polymers onto rotating tubular collector. (b2) Sheets produced by cells or decellularized tissue rolled onto mandrel. (b3) Hollowed tubular vessel fabricated by casing materials into annular mold. (b4) Implantation of rods into a body, subsequently encased by autologous tissue. Autologous hollowed conduits can then be obtained by explanting and removing the rod from surrounding tissue. Finally, (c) xenogeneic or allogeneic vessel decellularization can also be used as a source of vessel graft. Non-vascular tissues after decellularization can be rolled into tubular structure (through sheet-rolling) to form tubular vascular graft. (A portion of this figure was created with BioRender.com.)

Extrusion-based: in this method, a pneumatic or mechanical (piston or screw) force is used to extrude the bioink through a nozzle forming a filamentous shape.^127^ Pressurized air displaces the material through a nozzle. Alternatively, a piston or screw connected to an electric motor displaces the bioink [Fig. 2(a1)]. An advantage of the extrusion approach is the capability of printing high viscosity bioinks at a higher cell density (>1 × 10^6^ cells/ml or even cell spheroids) compared with the other 3D printing modalities.^126,128^

Large tubular structures are been obtained by extruding hydrogels in a concentric pattern in the vertical plane [Fig. 2(a2.1)] although shrinkage induced by cross-linking process is a limitation of this strategy.^129^ Development of tubular structures with multiple concentric layers resembling native blood vessel microarchitecture is difficult to achieve when spiral extrusion is performed in a vertical plane, but only short TEBVs can be obtained. To address this limitation, deposition on the surface of a rotating horizontal rod is used [Fig. 2(a2.3)]. For instance, Freeman *et al.* extruded neonatal human dermal fibroblasts, gelatin, and fibrinogen on the surface of a rotating rod.^130^ After the construct is submerged in thrombin solution and cultured up to 60 days without perfusion, a burst pressure of 1110 mm Hg is obtained. It is worth noting that the addition of perfusion stimulus should improve the mechanical properties.^130^

Vertical and horizontal 3D bioprinting with a rotating rod are suitable for simple hollow-tube development. However, they present some limitations when more complex branched structures with different diameters are needed. In an alternate approach, termed freeform reversible embedding of suspended hydrogels (FRESH) [Fig. 2(a2.2)], a bioink solution is extruded at 20 °C into a secondary hydrogel support bath, also known as fugitive ink, which provides mechanical support maintaining structural integrity and avoids spreading during the printing process. Raising the temperature to 37 °C melts the hydrogel support bath, leaving behind only the 3D printed construct.^131^ Using gelatin microparticles as support bath, bifurcated tubes are developed using different bioinks such as alginate, collagen type I, and fibrin. Moreover, a more complex structure such as branched coronary artery wall thickness of <1 mm and inner diameters ranging from 1 to 3 mm could be printed with alginate. A coronary artery-size vessel printed with collagen type I and perfused during 5 days with murine C2C12 cast around the structure demonstrated high cell viability and active remodeling of the construct.^132^

A novel strategy to form hollow micro-tubular constructs in one step is to adapt 3D printing with a coaxial nozzle [Fig. 2(a3.1)]. Coaxial nozzles usually have an inner core where the cross-linking agent is extruded and allows a rapid cross-linking of the outer shell hydrogel, resulting in stable hollow microtube structure.^133,134^ Cross-linking the hydrogel in two directions is achieved by adding an extra outer shell in the coaxial nozzle.^135^ The dimensions of the tubular constructs are determined by the dimensions of the core–shell nozzles. An advantage of this approach is that long hollow conduits can be obtained within a short period of time. Using this technique, vessels are derived in a one-step procedure utilizing a blend of ECFCs with alginate and vascular-tissue-derived decellularized extracellular matrix.^124^ When implanted in a mouse ischemic limb model, increased neovascularization and recovery of the ischemic limb occur. Using a triple coaxial nozzle with a sacrificial polymer in the inner core (e.g., Pluronic^®^), and a blend of alginate with collagen or collagen as outer and middle layers, results in a dual layered vessels. Human aortic SMCs and ECs can be encapsulated in the outer and inner layers, respectively, demonstrating native cell alignment without perfusion stimulus.^136^ Moreover, these vessels demonstrate functionality after *in vivo* implantation as an abdominal aorta graft, with a good patency and integration with the host tissue.^137^ When these vascular constructs are organized in 3D structures, scaffolds with vasculature can be directly developed, successfully supporting proliferation and maturation of vascular cells^134^ [Fig. 2(a3.2)]. When printing on the surface of a rotating rod, Gao *et al.* printed two layers encapsulating three different cell types: fibroblasts on the outer layer, smooth muscle cells in the middle layer, and HUVEC cells seeded on the surface of the inner layer, mimicking the native blood vessel architecture and reporting a cell viability over 90%.^123^

**FIG. 3.**
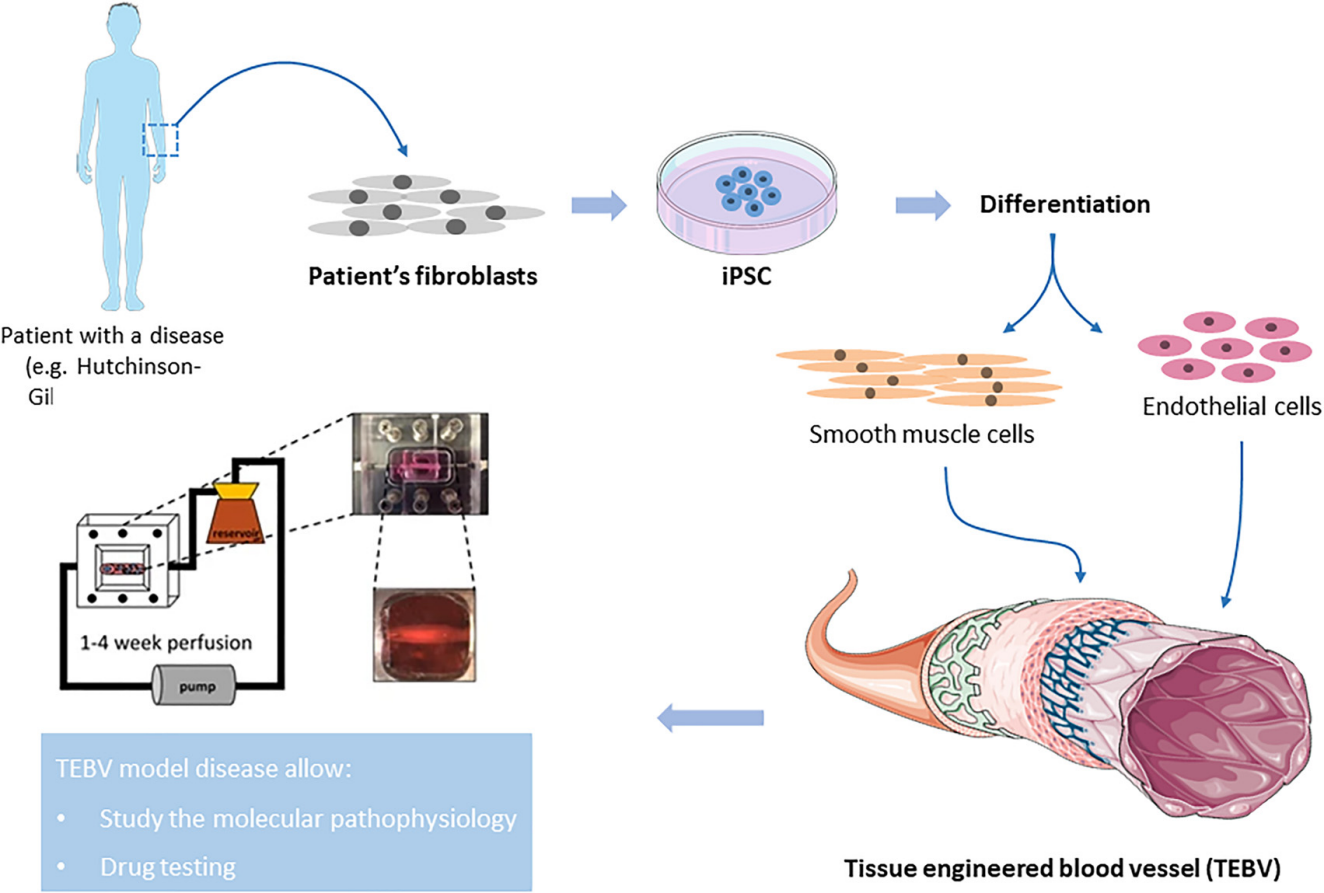
Overview of *in vitro* TEBV for patient-specific disease modeling. Somatic cells (for example, fibroblasts) from a patient with specific disease are reprogrammed to iPSC. Then, iPSCs are differentiated to vascular cell types, such as endothelial cells or smooth muscle cells, which are incorporated within tissue engineered blood vessel (TEBV). These TEBVs are able to recapitulate patient's disease phenotype, thereby, establishing a vascular model disease. Part of the figure is the images from Servier Medical Art (http://smart.servier.com/). Part of the figure is adapted from Atchison *et al.*, Sci. Rep. **7**(1), 8168 (2017).^171^ Copyright 2017 Authors, licensed under a Creative Commons Attribution (CC BY) license.

Inkjet bioprinting, also known as drop-based bioprinting, involves drop-by-drop bioink deposition with the use of thermal or piezoelectric forces.^126^ Pulses of pressure are generated by heating the print head, inducing the generation of droplets, whereas in piezoelectric inkjet, the bioink breaks into droplets due to acoustic waves generated by a piezoelectric crystal. Low viscosity bioinks avoid nozzle clogging but must quickly cross-link to form a solid structure after printing. This requirement limits the bioinks to alginate and its composities.^138^ Similar to the extrusion method, single tubular structures are obtained when a circular printing pattern is applied in the vertical axis.^139^ A hydrophobic and high-density fluid support, such as fluorocarbons, which do not mix with the printing material and preserve the printed structure, enables creating branched tubular structures.^140,141^ To overcome the droplet impact force, gravity, and buoyant forces^142^ non-circular printing enables printing of tubular shapes.^143^

Lacking any intrinsic cell adhesion sequences, alginate is limited in terms of enabling cell spreading. As an alternative, Schöneber *et al.* reported an *in vitro* blood vessel model with tri-layered structure using inkjet 3D bioprinting along the horizontal axis. Sacrificial gelatin with HUVEC was printed as the inner core followed by a deposition of fibrin and SMCs as tunica media. Finally, casting a hydrogel containing fibroblasts, fibrin and collagen around the previous structure formed the tunica adventitia. Perfusion with physiological flow rates results in functional TEBVs expressing VE-cadherin, smooth muscle actin and an increase in collagen type IV deposition, with a monolayer of ECs preserving the barrier function.^144^

#### 2. Electrospinning

Electrospinning is commonly used to create nano- and microscale fibers [Fig. 2(b1)]. The size range of these fibers is similar to the size of protein fibers in native ECM (50–500 nm).^145^ A polymer solution is loaded into the syringe and a droplet forms at the tip of the needle attached to the syringe due to surface tension. Then a high voltage is applied to the needle and once the electric force overcomes the surface tension, a Taylor cone form at the tip of the needle. The charged polymer jet travels several cm, during which the solvent evaporates and the resulting polymer fibers land on a collector. The collector is either stationary or rotating to form randomly aligned fibers or aligned fibers, respectively.

Electrospinning is widely used to fabricate TEBVs with synthetic materials, such as PCL, PGA, poly(l-lactide-co-ε-caprolactone) (PLCL), poly(ester urethane) urea (PEUUR), natural materials such as silk fibroin, or the mixture of both. The concentration of the polymer, rate of extrusion, distance between the needle and collector, types of collector and collector rotation speed affect the final shape, fiber size, pore size, and properties of the final product.^146–148^ A bi-layer vascular graft is obtained by electrospinning PCL-silica as the outer layer and PCL-collagen as the inner layer, exhibiting enhanced mechanical strength relative to PCL grafts.^149^

#### 3. Sheet-rolling

The sheet used with this method [Fig. 2(b2)] is generated from different sources including synthetic and natural polymer materials, and a sheet is formed by a confluent cell monolayer.^78,150,151^ The sheets are rolled on the surface of a tube or a mandrel, which is then removed. Sheet-rolling can be combined with electrospinning to create a graft mixed with PEUUR and fibrin.^152^

Instead of using scaffolds as supports, TEBVs can be produced by rolling a confluent sheet of SMCs around a mandrel followed by another sheet of fibroblasts. Then the mandrel is removed and the inner lumen is seeded with ECs.^50^ After maturation for 3 months, the burst pressure is over 2000 mm Hg. While this approach limits the amount of ECM, the fabrication process is rather long.

#### 4. Mold-shaping

Mold-shaping (or casting) [Fig. 2(b3)] was used by Weinberg and Bell, the first to manufacture a TEBV that mimicked the adventitia, media, and intima layers of a vessel. Generally, using this method, collagen with SMCs or other medial cells is cast into an annular mold. After removing the construct from the mold, a structural support is needed^86^ since the burst pressure is around 100 mm Hg (Ref. 153) due to low mechanical properties of collagen gels. In contrast, fibrin gels seeded with cells and decellularized afterwards could be implanted as a pulmonary artery in lambs.^99,154^ After 10 months, the grafts remain patent with good host cell integration and matrix deposition. Instead of casting gel/cell mixture into the mold, Kelm *et al.* first made tissue droplets with fibroblasts by the hanging-drop method to produce micro-tissue with fibroblasts secreted and later placed these droplets into molds. The micro-droplets remodeled in 14 days and positive luminal α-SMA expression was observed indicating myofibroblast differentiation.^155^

#### 5. In vivo autologous TEVS formation

Autologous grafts [Fig. 2(b4)] can be fabricated by placing silastic tubing (3 or 5 mm outer diameter) into the peritoneal cavity of an animal forming multilayers of myofibroblasts and a single layer of mesothelium in about two weeks. Using this approach, grafts achieve burst pressure above 2500 mm Hg.^156^ Although this method is promising as an *in vivo* replacement graft, there are discrepancies in the vessel structure and cell composition when compared to native vessel. The translation of these TEBVs to humans has not been reported yet.

**FIG. 4.**
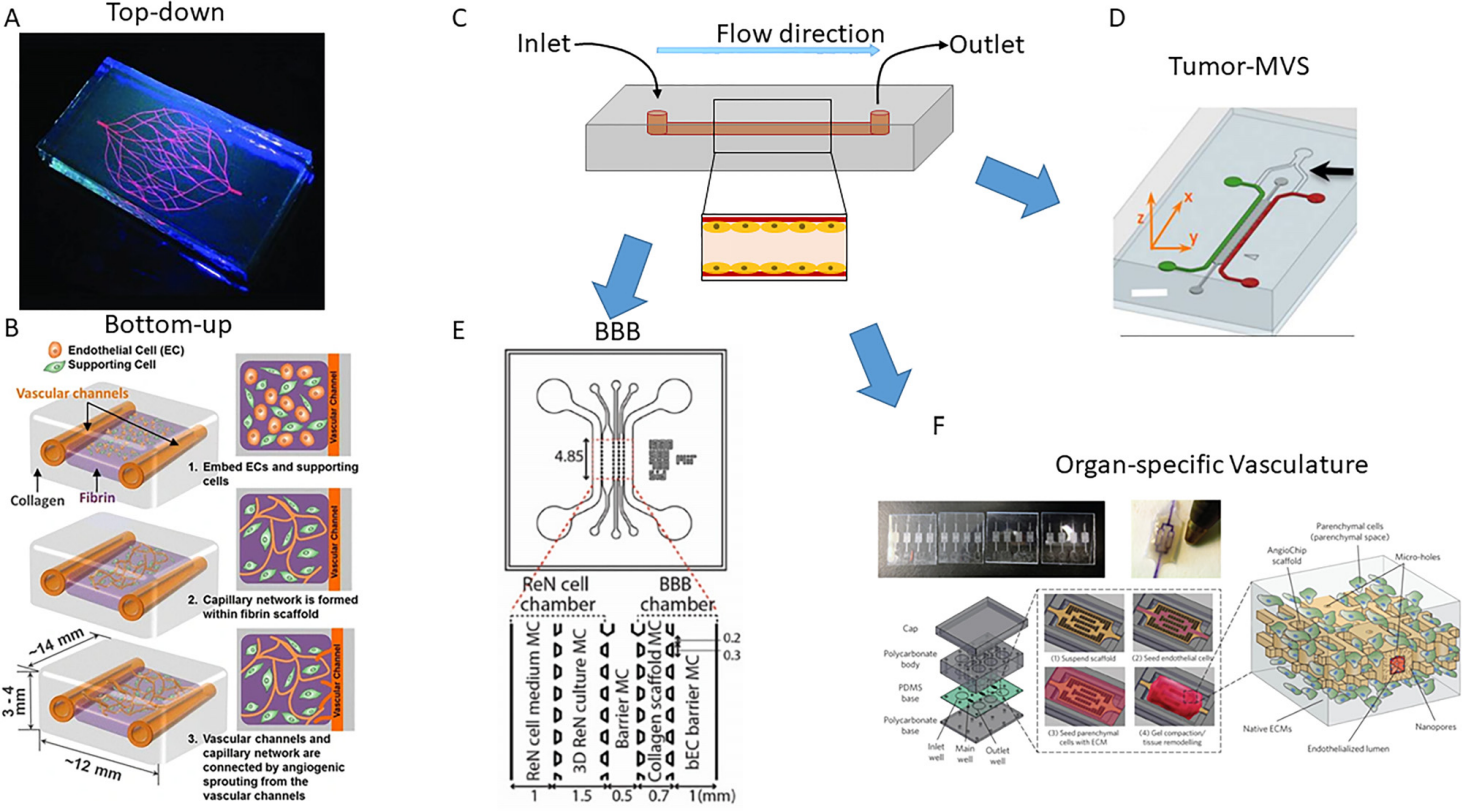
Biofabrication approaches and applications for microvascular systems. (a) Top-down approach: predesigned microvascular systems were fabricated by direct or indirect three-dimensional bioprinting techniques. Picture is reprinted with permission from Wu *et al.*, Adv. Mater. **23**(24), H178–H183 (2011).^186^ Copyright 2011 Wiley‐VCH Verlag GmbH & Co. KGaA, Weinheim, John Wiley and Sons. (b) Bottom-up approach: endothelial cells are seeded into gel matrix to form the new capillary network by inducing vasculogenesis or angiogenesis through native physiological cues. Reprinted by permission from Lee *et al.*, Cell. Mol. Bioeng. **7**(3), 460–472 (2014).^190^ Copyright 2014 Springer Nature Customer Service Center GmbH, Biomedical Engineering Society. (c) Schematic representation of a simple microfluidic device. Fluid flow enters from the inlet and leaves from the outlet. Endothelial cells are seeded in the channel. (d) MVS can be used to study the progression of cancer by co-culturing cancer cells (red channel) with ECs and stromal cells (green channel). Reprinted with permission from Zervantonakis *et al.*, Proc. Natl. Acad. Sci. **109**(34), 13793–13798 (2012).^205^ Copyright 2012 The Proceedings of the National Academy of Sciences (PNAS). (e) MVS seeded with neural cells (ReN) and endothelial cells (bEC) can be used to model BBB for understanding the underlying mechanisms or for treatment discoveries for neurological disorders and Alzheimer's disease. Picture is adapted from Shin *et al.*, Adv. Sci. **6**(20), 1900962 (2019). Copyright 2019, Authors licensed under a Creative Commons Attribution (CC BY) license. (f) MVS can be incorporated into other organ system to supply oxygen and nutrients or to study the interaction of parenchymal cells and endothelial cells. Reprinted by permission from Zhang *et al.*, Nat. Mater. **15**(6), 669–678 (2016).^233^ Copyright 2016 Nature Publishing Group.

#### 6. Decellularized tissue

Decellularization of tissue [Fig. 2(c)] provides a source material for the scaffold and an “off-the-shelf” solution for the clinical use of TEBVs. An ideal decellularization method for fabricating TEBVs should yield scaffolds that preserve the composition and mechanical properties of original tissue yet completely remove the original cellular and nuclear materials to avoid adverse immunoreaction. Typical methods used to decellularize blood vessels are with enzymes (trypsin with EDTA)^157^ or detergents (Triton-X or CHAPS and sodium dodecyl sulfate).^100,158–160^ Decellularization with detergent or hypotonic solution does not greatly compromise the mechanical properties of the original vessel.^161^

Common sources for decellularized tissues are from porcine or bovine vascular or non-vascular tissues. Decellularized grafts have shown great success as grafts for *in vivo* application in small, large, non-human primate animal models and even human trials.^80,100,159,162–169^ Bader *et al.* decellularized porcine aortas and subsequently seeded human EC and myofibroblasts isolated from saphenous vein biopsy.^162^ The matrix can be re-endothelialized and SMCs migrated into the matrix. To assess the immune response, decellularized grafts implanted in rats had less CD8 T cell infiltration compared to unmodified human saphenous vein controls. Grafts prepared from decellularized porcine iliac vessels and seeded with ECFCs isolated from peripheral blood of sheep remained patent after implantation for 130 days.^158^ Decellularized porcine small intestine submucosa (SIS) has been tested widely as a valid source for fabricating TEBVs. After implantation for 180 days in the carotid artery in a canine model, SIS grafts outperformed ePTFE grafts in overall patency rate (7/8 SIS vs 2/8 ePTFE), re-endothelialization, and neovasa vasorum formation.^163^ Immobilization of heparin and vascular endothelial growth factor (VEGF) onto SIS in an ovine model resulted in a fast endothelialization rate 1-month post-implantation and 92% patency rate over 3 months.^164^

Decellularized bovine scaffolds have been commercialized and compared to standard synthetic polymer grafts like PTFE^165,166^ or autologous vein.^167^ In a recently published clinical study of bovine carotid artery graft (Artegraft) was compared to autologous vein^167^ as bypass or interposition in traumatic arterial injuries. Although the autologous veins had slightly better patency rate (primary: 85% vs 78%, secondary: 100% vs 78%) and limb salvage (94% vs 82%), the bovine carotid artery graft is a viable choice while veins are not available. Another clinical study of bovine carotid artery as lower leg bypass graft showed a five-year primary patency of 67.5% and secondary patency of 75.6% and limb salvage rate.^168^

The first human trial with an allogeneic decellularized scaffold was performed by Olausson *et al.* in 2012^159^ who used a decellularized 9 cm segment of iliac vein from a deceased donor and recellularized it with autologous ECs and SMCs derived from bone marrow stem cells. Even though another procedure was required to lengthen the graft with another vein graft due to the narrowing of the graft at 1 year, the patient performed well and did not require any immunosuppression treatment.

Dahl *et al.*, isolated human aortic SMCs and seeded them into PGA scaffolds (6 mm ID) for 7 to 10 weeks to mature.^80^ The mature scaffolds are then decellularized and stored long-term at 4 °C. Autologous ECs were isolated and seeded into the scaffold before implantation. Following peripheral and coronary artery bypass in a canine model and arteriovenous access for hemodialysis in a non-human primate model, the mechanical properties, host cell infiltration, and patency rate were promising. In a similar approach, Syedain *et al.* first fabricated 4 mm grafts by mixing neonatal human dermal fibroblasts with bovine fibrin gel,^100^ which matured in 5 weeks. The grafts were decellularized with sodium dodecyl sulfate (SDS) and Triton X-100 and stored for up to 12 months. Arteriovenous grafts in baboons showed no calcifications and stenosis after 3 and 6 months with patency rates of 83% and 50%, respectively. Although promising, more effort is needed to improve the patency rate of these vessels.

TEBVs made of decellularized tissue closely resemble the ECM microenvironment of the native vessel and possess great mechanical and biological properties. A major advantage of decellularized grafts is long-term storage to make them readily accessible for clinical use. These features enable the decellularization approach for fabricating TEBVs for *in vitro* applications.

### D. Disease models and *in vitro* applications of TEBV

*In vitro* TEBV systems serve as an alternative to animal models. In addition to the necessary requirement for TEVS noted before, there are three more features which should be considered when choosing methods and materials for fabricating TEBVs for *in vitro* disease modeling purposes. First, the time for overall fabrication and maturation should be within one week. Second, the TEBV should maintain measurable vasoactivity, including vasodilation and vasoconstriction. For many vascular diseases, endothelial dysfunction is one of the earliest events and is manifest by reduced vasodilation.^93^ Third, cells used for fabricating the TEBVs should have disease phenotypes, ideally isolated from patients with the disease being modeled. Advances in stem cell technology, especially the development of iPSCs,^32,170^ enable fabrication of TEBV that can model diseases and testing treatment response (Fig. 3). SMCs have been derived from iPSCs of individuals with progeria,^171^ supravalvular aortic stenosis,^172^ and Marfan syndrome.^173^ By integrating iPSC-derived SMCs and ECs into TEBVs, they can recapitulate the phenotypes developed with the disease and be used as a platform to test treatment responses.

A TEBV model using iPSC-derived SMCs from individuals with Hutchinson–Gilford progeria syndrome (HGPS) recapitulated many features in HGPS such as reduced vasoactivity, increased medial wall thickness, and increased calcification and apoptosis of SMCs.^171^ In addition, this TEBV model showed that the treatment with Everolimus drug removes progerin.^171^ A follow-up study included HGPS iPSC-derived EC into the system and showed reduced TEBV function and EC dysfunction markers.^174^ By fabricating the layers of the vessel with different cell combinations (HGPS-EC with normal SMC, normal EC with HGPS-SMC, etc.), they separated the contributions of each cell type. These TEBVs can be used to examine the effect of genetic variations on drug responses and develop personalized treatment solutions or solutions for specific populations.^175^

Atherosclerosis is one of the major underlying cause for CVDs. Accumulation of foam cells and forming cholesterol plaque can eventually lead to ischemia and stroke. Human immune system and inflammation play key roles in the initiation and progression of atherosclerosis. To model atherosclerosis *in vitro*, Zhang *et al.* fabricated a TEBV system by plastic compression of collagen gel.^49^ The TEBV has either two-layer (EC and fibroblast) or three-layer (EC, SMC, and fibroblast) and closely resemble the structure and mechanical properties of native vessels. They showed that enzyme modified LDL and pro-inflammatory cytokine TNF-α synergistically promoted the activation of endothelial cells and subsequent accumulation of monocytes. They further demonstrated the suitability as a drug testing platform by showing that lovastatin or NF157 (a P2Y11R antagonist) reduces monocyte accumulation and foam cell formation. In another study, Gao *et al.* used 3D in-bath coaxial cell printing with ECM from decellularized porcine aortic tissues and sodium alginate as bioink to fabricate a triple-layered engineered blood vessel for modeling early stage of atherosclerosis.^176^ This system could recapitulate the hallmarks of early stage atherosclerosis such as endothelial activation, macrophage adhesion and differentiation, LDL accumulation, and foam cell formation. They further demonstrated the capability of this model to be used as drug testing platform by showing the dose dependent effect of atorvastatin on inhibiting foam cell formation.

Thrombotic disorders caused by disruption of the balance between procoagulant and the anticoagulant factors lead to pathologic formation of thrombi in vascular systems and may cause stroke and limb ischemia.^177^ Many vascular microphysiological systems (MPS) have been developed to model the thrombotic disorders and reveal the underlying mechanism by which thrombotic disorder is developed. Zhang *et al.* used the sacrificial bioprinting technique to create microchannels with Pluronic F-127 (sacrificial mold) and subsequently seeded HUVECs in gelatin methacryloyl hydrogel. Then, they perfused human whole blood and induced thrombi formation in the vessel and could prove that tissue plasminogen activator dissolves non-fibrotic clots.^178^ Barrile *et al.* fabricated a microengineered blood “vessel” system made of polydimethylsiloxane (PDMS) with a channel covered with confluent endothelial cells.^179^ They show that prothrombotic effect of Hu5c8-IgG1, a monoclonal antibody that targets CD40L for treating of autoimmune disorders and causes thromboembolism as side effect, is due to the interaction of IgG1 and FcyRIIa receptor on platelets, which activates platelets. Hu5c8-IgG2σ, a revised version of the antibody that does not interact with FcyRIIa receptor, did not induce significant increase in platelet aggregation and fibrin deposition compared to control.

In addition to disease modeling, the TEBVs also serve as a platform for drug toxicity and efficacy testing. A collagen based TEBV system was seeded with cord blood or Coronary Artery Disease (CAD) patient blood-derived endothelial colony forming cells and human neonatal fibroblasts.^93^ Its perfusion with a nitric oxide synthase inhibitor blocked the vasodilation to acetylcholine, caffeine and theophylline. These TEBVs also showed acute activation of endothelium by TNF-α which was blocked by the statin lovastatin. One future application is to incorporate TEBV disease model to other MPS system like myobundles or cardiac bundles to study the process of drug release or immune cell accessibility.

Although the current generation of TEBVs can as an *in vitro* platform for disease modeling and drug screening, the TEBV systems can be further improved. Future efforts are needed to identify the most appropriate materials that could produce similar mechanical properties to the nature vessel and better reproduce the medial structure. While iPSC-derived SMCs demonstrate suitable contractility, methods to increase elastin need to be developed. One other direction is to incorporate TEBV with other body systems such as the immune system and the pulmonary system.

## III. FABRICATION OF MICROVASCULAR SYSTEMS

The MVS consists of arterioles, venules, and capillaries. Capillaries are networks of micrometer-size blood vessels in which exchange of gases (oxygen to carbon dioxide) and nutrients/wastes take place. Instead of forming a single tubular structure, capillaries form networks. The wall of most capillaries consists only of a layer of endothelium and experience much lower pressure than arteries.^180^ These structural and functional differences between capillaries and large vessels require different fabrication approaches. Two strategies are used to fabricate microvascular systems: top-down and bottom-up. With top-down approaches, the detailed structure of the microvascular system is predesigned and endothelium forms around the pre-defined route. In bottom-up approach, endothelial cells, pericytes or pericyte-like cells are first mixed with hydrogels. Then, chemical or mechanical cues are applied to the system to promote angiogenesis and/or vasculogenesis of pre-seeded cells.

### A. Methods and materials for MVS fabrication

#### 1. Bioprinting

Bioprinting techniques have been widely used to fabricate microvascular systems and can be categorized into direct and indirect approaches.

Direct-printing utilizes quick gelation properties of bioink or prints bioink into solution to print a stable pre-designed structure. For instance, Bertassoni *et al.* bioprinted agarose gel and then embedded into photocrosslinkable hydrogel (GelMA). After the cross-linking of the hydrogel and agarose removal, the fabricated hollow microchannels, 150 *μ*m in diameter,^181^ formed individual tubes instead of an interconnected system. Grigoryan *et al.* used stereolithography with monolithic hydrogels [water and poly(ethylene glycol)] diacrylate to bioprint perfusable complex entangled vascular networks, which allows intervascular oxygen transport. They incorporated food dyes as photoabsorbers to enhance the z resolution, which improved pattern fidelity and advanced the architectural richness.^182^ The resolution of laser-assisted direct extrusion (∼200 *μ*m),^183^ bioprinting,^184^ and inkjet printing^185^ prevents printing of capillary size vessels.

With indirect printing, sacrificial materials (e.g., Pluronic^®^, alginate, gelatin, agarose, amongst others), which are dissolved later by thermal modifications or chemical reactions, are first printed into a pre-designed structure and encapsulated into hydrogel. Then, the sacrificial materials are dissolved and leave behind perfusable microvascular networks, where ECs are seeded latterly. As one example, Wu *et al.* deposited aqueous Pluronic F127 as sacrificial ink for a pre-designed microvascular pattern in diacrylated Pluronic F127, which has a higher critical micelle concentration than pure Pluronic F127, producing perfusable microvascular system with the smallest channel diameter of 150 *μ*m.^186^ Later, Miller *et al.* used 3D thermal extrusion printing with glucose–sucrose–dextran ink to generate plastic lattices with predesigned 3D structures.^187^ Because these lattices are stiff, they are suitable as sacrificial molds in matrices gelled by different mechanisms, such as chelation (sodium alginate), photopolymerization (GelMA), enzymatic (fibrin), and thermal (collagen and Matrigel). Other commonly used bioink/base-matrix pairs are Matrigel/collagen,^188^ gelatin/collagen or fibrin,^189,190^ and Pluronic F127/gelatin methacrylate.^191^ Ice can be cast and 3D-printed to form sacrificial materials with different architectures, which meet the need for fabricating vessels with different shapes.^192^ In an effort to create pulmonary arterioles, Jin *et al.* used a sacrificial germanium layer to seed ECs and SMC layers which were then folded into tubes with lumen.^193^ EC in these microvessels produce significantly more nitric oxide (NO) than cells cultured on a flat surface.

Both the direct and indirect printing studies mentioned here followed the top-down [Fig. 4(a)] approach by fabricating MVS with predesigned architectures. With this approach, complex MVSs could be designed to study the physical and biological mechanisms of material/gas through the capillary network with well-designed architectures. However, the current techniques do not have enough resolution to bioprint MVSs on a capillary scale.

Bottom-up or self-assembly approaches [Fig. 4(b)] are based on the native physiological cues that induce vasculogenesis or angiogenesis to drive the formation of the new capillary network. Bottom-up approach allows the formation of capillary vessels with size similar to native capillary. Vasculogenesis and angiogenesis are driven by both mechanical (flow) and chemical cues.^205^ Lee *et al.* developed a three-dimensional system^190^ by printing two parallel gelatin tubes as sacrificial material onto a collagen layer. After that, they deposited a layer of fibrin containing HUVECs and normal lung human fibroblasts (NHLFs) between the two gelatin tubes and finally printed several layers of collagen on top of the gelatin tubes and fibrin layer. The gelatin tubes were then dissolved, which left two hollow channels into which HUVECs were seeded. A peristaltic pump was used to create fluid shear stress. Angiogenesis was observed in the hollow tube and HUVECs embedded in the fibrin gel were able to form capillaries with lumen after 14 days.^190^ In a recent study, Palikuqi *et al.* introduced the E26 transformation-specific (ETS) variant transcription factor 2 (ETV2) into mature ECs which were then seeded in fibrin gel.^194^ These cells could form blood-perfusable multilayered branching networks within three days. These approaches show great promise, but one of the major challenges with bottom-up approaches is the control over the flow rate in individual vessels.

#### 2. Microfluidic device

The introduction of microfluidic devices allows application of controllable mechanical stresses to cells through fluid flow and pressure drops. These stimuli mediate EC functional phenotype^195^ and proliferation,^196^ and stimulate angiogenesis to split a single vessel into two or more vessels.^197^ A typical microfluidic device consists of inlets, conduct channels, and outlets [Fig. 4(c)]. The device is first designed using a computer-aided design program and then cast to mold (for example, silicon wafer) by soft photolithography. Polydimethylsiloxane (PDMS) is used to create the base mold and adhere onto glass or plastic culture dish. The inlet and outlet can be created by biopsy punches. Flow driving force is achieved by pumps or hydrostatic pressure. To mimic the microenvironment, biological materials such as collagen and fibrin are used in microvascular microfluidic devices for cell growth and angiogenesis.

Song and Munn fabricated a three-channel microfluidic device. The two outer layer channels are covered with HUVECs separated by a collagen gel to show that fluid stresses on ECs attenuate their sprouting.^198^ Viscous fingering is another approach to form hollow channels by using flow of less viscous fluid (phosphate buffered saline, for example) to displace a more viscous solution, such as collagen by flow to fabricate a vessel-on-a-chip with arteriole size vessels (∼261 *μ*m).^199^ Moya *et al.* fabricated a microfluidic device to create an interconnected capillary network by driving vasculogenesis of human ECFCs embedded in fibrin gel with human normal lung fibroblasts (NHLFs) through interstitial flow.^200^ These microvascular systems have a filtration limit similar to human capillaries. Mixing the colorectal cancer cell line HCT116 with ECFCs and NHLFs in arrays of vascularized micro-organs on a standard 96-well plate,^201^ they identified anti-tumor and anti-angiogenic drugs among a panel of 10 different Food and Drug Administration (FDA)-approved drugs in a blinded study. The versatility of microfluidic device also allows researchers to study the role of growth factors and cell-to-cell interactions in early vasculogenesis and angiogenesis.^202,203^

### B. MVS disease modes and *in vitro* applications

MVSs are widely used in tumor biology to study angiogenesis, tumor progression, the tumor microenvironment,^204–209^ the blood–brain barrier (BBB), and diseases that affect the microvasculature such as sickle cell disease, or even to investigate the biophysical and biomolecular interaction of malaria-infected erythrocytes.^210^

#### 1. Cancer model

Many features of the *in vivo* cancer growth environment can be recapitulated *in vitro* by co-culturing cancer cells with stromal cells and supplying necessary molecules and nutrients through microfluidic channels. In addition to ECs, tumor vascularization involves pericytes, macrophages, cancer associated fibroblasts (CAF), myeloid-derived suppressor cells, and mesenchymal stem cells.^211^ CAFs and macrophages recruited to the tumor microenvironment secrete vascular endothelial cell growth factor and promote tumor vascularization.^211^ Furthermore, vessels in the tumor microenvironment are essential for drug delivery and to allow immune cell access. Tumor-MVSs are fabricated by co-culturing cancer cells with ECs and stromal cells [fibroblast, etc., Fig. 4(d)]. After designing the structures of the channels and seeding with ECs, the tumor cells are well mixed prior to injection in the gel chamber. To study the stages during cancer progression, tumor cells are placed in a different channel adjacent to ECs.

Zervantonakis *et al.* fabricated a chip with an EC channel formed by microvascular ECs [Fig. 4(d), green channel] and a tumor channel with fibrosarcoma cells or breast cancer cells [Fig. 4(d), red channel], separated by an ECM layer [Fig. 4(d), dark gray channel].^205^ Macrophage-secreted TNF-α enhanced endothelial permeability and the cancer cell intravasation rate. Tumor-MVSs also allow easy access for high resolution microscopy and live-time monitor of tumor–EC interactions, which is a challenge with *in vivo* animal studies.^212–214^ Lee *et al.* created a metastasis chip with a clear EC–cancer cell boundary, which allows high resolution imaging to monitor the angiogenic response and EC–cancer cell interactions.^215^ This chip reproduces the response of cancer angiogenesis to the anti-VEGF drug bevacizumab. By co-culture of cancer cells with ECs and fibroblasts in fibrin gel, Ehsan *et al.* modeled the early cancer cell–EC interaction of solid tumor. The Slug transcription factors involved in the epithelial to mesenchymal transition influenced how the increase in intravasation depended upon low oxygen conditions.^216^ Using a three-channel system with two outside channels as a cancer cell channel and an inner perfusable microvascular channel containing myoblasts to mimic muscle microenvironment or osteo-differentiated stem cells to mimic bone microenvironment, Jeon *et al.* were able to identify the role of adenosine in cancer cell extravasation. They showed that higher cancer cells extravasation rates are induced by blocking A_3_ adenosine receptors with antagonist in muscle microenvironment and supplying adenosine to bone microenvironment reduced the cancer cell extravasation.^217^

#### 2. Blood–brain barrier

The blood–brain barrier (BBB) is a special micro-vascular system formed by a layer of brain vascular endothelial cells surrounded by pericytes in an ECM protein rich basement membrane. Unlike capillary networks in other tissues, endothelial cells in BBB form tight junctions between cells and tightly control the material transport between blood and brain through interaction with other cells in the brain such as pericytes, astrocyte, and microglia. BBB function is altered with aging. For example, the capillary wall thickness increases and the expression of tight junction proteins decreases during aging.^218^ Dysfunction of BBB is associated with neurodegenerative pathologies such as Alzheimer's disease (AD) and Parkinson's disease.^218–220^

Microvascular systems have been widely used to model the BBB as an alternative to the use of animal models^221^ for understanding the underlying mechanisms or for drug developments^222,223^ [Fig. 4(e)]. The BBB has been modeled with MVSs^224^ to study neurological disorders^225^ and Alzheimer's disease^226^ using primary cells or iPSCs. To model Alzheimer's disease (AD), Shin *et al.* combined a BBB chamber seeded with human cerebral microvascular endothelial cells, forming tight monolayer at the interphase of collagen scaffold with a cell chamber with either wild type (WT) ReNell VM human neural progenitor (ReN) cells or ReN cells with familial Alzheimer's disease mutations in *APP* gene and *APP*/*PSEN*1 genes.^226^ This system could recapitulate many features of BBB dysfunction in AD patients including increased BBB permeability, loss of tight junction protein expressions, increase level of MMP-2 and reactive oxygen species (ROS), and accumulation of beta-amyloid peptides at the endothelium. Vatine *et al.* incorporated patient-specific iPCSs in their BBB-chip to create a platform with physiologically relevant transendothelial electrical resistance and permeability. Their platform detected alterations in BBB function among different patients. Furthermore, perfusing blood with 3 kDa dextran showed limited permeability of dextran.^225^ To replicate more closely the *in vivo* BBB, Campisi *et al.* induced vasculogenesis of ECs seeded in fibrin gel with pericytes and astrocytes, fabricating a perfusable mature BBB MPS that was suitable for modeling neurodegenerative diseases and drug development.^227^ To increase the drug screening throughput, Zakharova *et al.* fabricated a BBB-chip with eight channels, which allowed running of eight parallel tests simultaneously in one single chip.^228^

#### 3. Organ-specific vasculature

One of the major challenges for tissue culture and organs-on-a-chip is the diffusion limit of oxygen and nutrient supply, which is around 200 *μ*m.^229^ Hence, re-vascularizing engineered tissue and organ-on-a-chip systems is essential for the survival of cells in these systems, especially for systems that mimic organs with well-vascularized network such as liver and kidneys [Fig. 4(f)]. MVSs have been incorporated into other organ systems such as lung,^230^ liver,^231–233^ kidney,^234^ and islets^235^ to supply oxygen and nutrients or to study the interaction of parenchymal cells and endothelial cells in MVS.

Zhang *et al.* built an AngioChip using biodegradable material poly(octamethylene maleate (anhydride) citrate) with internal channels for EC vasculogenesis and outer space for hydrogel containing parenchymal cells to encapsulate the internal channels.^233^ The internal channels of EC formed a vascular network and the hydrogels formed tissue systems based on the type of parenchymal cells. Using this method, they successfully fabricated functional vascularized liver and millimeter-scale functional cardiac tissue. Ligresti *et al.* developed a kidney MVS to model nephrotoxicity and progression of kidney disease.^234^ Incorporation of microvascular meshes to islets and their transplantation to subcutaneous tissue in rats enhanced recovery of chemically induced diabetes.^235^ With iPSC derived ECs, MVSs could be further used to model microvascular diseases or serve as a model for testing gene-therapies.^236,237^

## IV. CONCLUSION AND FUTURE PROSPECTS

The development of TEVS addresses a critical gap between high demands of vascular graft and shortages of available autologous arteries or veins. Success with large diameter vessels has proven the feasibility of using TEBV in clinical settings. In terms of small diameter grafts, many approaches that use different biofabrication techniques and biomaterials show promising results in pre-clinical studies. Although it is still too early to determine what is the best approach, understanding the mechanisms of each component of native vessels that contribute to the overall mechanical and biological properties would provide critical information for designing TEVS. Advances in biomaterials development and biofabrication techniques provide research necessary tools to fulfill the design requirement of TEVS. The ideal biomaterial should be biocompatible with strong mechanical strength and suitable biodegradation time. The surface of a biomaterial should allow adequate endothelial progenitor cell (EPC)/EC attachment but not the activation of platelets. An ideal biofabrication technique for fabricating TEVS should possess properties including time efficiency and high resolution. For *in vitro* applications of TEVS, in addition to the requirement of mechanically strong properties, the TEVS should resemble the key biological features of a native vessel, which include the vasoactivity, cell/protein compositions, etc. Given this requirement, systems made with largely biological materials are more suitable for developing TEVS for *in vitro* application.

Cells are critical components for the long-term function of TEVS. For bypass procedures, an endothelium layer, which could provide non-thrombotic surface and releases nitric oxide for flow-mediated vasodilation, and mural cells, including SMCs, mesenchymal stem cells (MSCs), and fibroblasts to provide contractility in response to flow, are required. The discovery and development of iPSCs provide a steady source to overcome the shortages in autologous cells. Although many studies have successfully derived iPSCs to ECs and SMCs, many of these cells are not mature. Hence, the differentiation process of iPSCs to obtain mature cells needs to be improved. One promising approach is to combine CRISPR with small molecule approaches to activate endogenous transcription factors.^35^ Creating small diameter TEVS that closely resemble native vessel architecture and vascular environment with iPSC could also be used as model systems for diseases and drug testing platforms. A key challenge is to promote elastin synthesis. While pulsatile stretching of TEVS and a mixture of SMCs and fibroblasts can enhance elastin production, elastin levels are still below those found in native arteries.^238^ Creation of an adventitial layer would allow examination of its role in immune functions.

The easy modification feature of TEVS allows researchers to separate various genetic factors and test them separately to find out their individual contribution to the overall disease state. In addition to mm scale of TEVS, MVSs could also be used to study angiogenesis^239,240^ and the blood–brain barrier.^241^ Future efforts in this field should focus on developing biomaterial and biofabrication techniques that can fabricate TEVS that more closely resembles the mechanical properties and ECM environment of native vessels with a shorter production period.

## AUTHORS' CONTRIBUTIONS

Q.Z. and E.B.-R. contributed equally to this work.

## Data Availability

The data that support the findings of this study are available from the corresponding author upon reasonable request.
